# Supplementary material for: Realization and topological properties of third-order exceptional lines embedded in exceptional surfaces
Source: Nat Commun. 2023 Oct 20;14:6660. doi: 10.1038/s41467-023-42414-z (PMC10589303; doi:10.1038/s41467-023-42414-z)
Supplement: Supplementary file 1 — Supplementary Information [file 41467_2023_42414_MOESM1_ESM.pdf]

Supplemental Information for

Realization and Topological Properties of Third-Order Exceptional Lines Embedded in Exceptional Surfaces

Weiyuan Tang[1,2], Kun Ding[3*], Guancong Ma[1*]

[1] Department of Physics, Hong Kong Baptist University, Kowloon Tong, Hong Kong, China

[2] Department of Physics, The University of Hong Kong, Pokfulam Road, Hong Kong, China

[3] Department of Physics, State Key Laboratory of Surface Physics, and Key Laboratory of Micro and Nano Photonic Structures (Ministry of Education), Fudan University, Shanghai 200438, China

Supplementary Note 1 Finding the EP3s using the resultants

The condition of EPs given by the vanishing of the EPs are found at the intersections of the surfaces defined by $\mathrm{Re}(\mathcal{D}) = 0$ and $\mathrm{Im}(\mathcal{D}) = 0$, where $\mathcal{D}$ is the discriminant of the characteristic polynomial $p(\omega) = \det(H_{3b} - \omega\mathbf{I})$. For our model,

$$p(\omega) = \omega^3 - M(\phi_2)\omega^2 + [L^2(\phi_1) - 2N^2(\phi_3)]\omega - L^2(\phi_1)M(\phi_2). \quad (1)$$

For convenience, we denote $a_3 = 1$, $a_2 = -M(\phi_2)$, $a_1 = L^2(\phi_1) - 2N^2(\phi_3)$, and $a_0 = -L^2(\phi_1)M(\phi_2)$. Then, we have

$$\mathcal{D} = (-1)^{N(N-1)/2}\mathcal{R}\left[p(\omega), p(\omega)^{(1)}\right] = (-1)^{N(N-1)/2}\det\left[\mathrm{Syl}[p(\omega), p(\omega)^{(1)}]\right], \quad (2)$$

wherein $N = 3$ for our system, $\mathrm{Syl}[p(\omega), p(\omega)^{(1)}]$ is the Sylvester matrix

$$\mathrm{Syl}\left[p, p^{(1)}\right] = \begin{pmatrix} a_3 & a_2 & a_1 & a_0 & 0 \\ 0 & a_3 & a_2 & a_1 & a_0 \\ 3a_3 & 2a_2 & a_1 & 0 & 0 \\ 0 & 3a_3 & 2a_2 & a_1 & 0 \\ 0 & 0 & 3a_3 & 2a_2 & a_1 \end{pmatrix}, \quad (3)$$

wherein a_i is the coefficients in $p(\omega)$ with $i = 0,1,2,3$. Explicitly,

$$\mathcal{D} = -L^6(\phi_1) + 8N^6(\phi_3) - 2L^4(\phi_1)M^2(\phi_2) - L^2(\phi_1)M^4(\phi_2) + 6L^4(\phi_1)N^2(\phi_3)$$
$$-10L^2(\phi_1)M^2(\phi_2)N^2(\phi_3) - 12L^2(\phi_1)N^4(\phi_3) + M^2(\phi_2)N^4(\phi_3). \quad (4)$$

Notably, due to PT symmetry, $\mathcal{D} \in \mathbb{R}$, i.e., we have $\mathrm{Im}(\mathcal{D}) = 0$ everywhere. Thereby, the codimension for EP2s can be reduced from two to one, while the codimension for EP3s

decreases from four to two, which enables the EP2s and EP3s to simultaneously occur in a three-dimensional synthetic space. When $\phi_2 = \pm\pi/2$, which means $M(\phi_2)$ vanishes, the coalescence of three eigenstates gives rise to an EP3[1]. Then we have

$$p(\omega) = \omega^3 + [L^2(\phi_1) - 2N^2(\phi_3)]\omega. \tag{5}$$

The discriminant $\mathcal{D}$ then vanishes at

$$L^2(\phi_1) - 2N^2(\phi_3) = 0. \tag{6}$$

This condition can also be derived from the formulas of complex eigenvalues of the Hamiltonian. When $\phi_2 = \pm\pi/2$, the three eigenvalues are

$$\omega_{1,3} = (\omega_0 - i\gamma_0) \pm \sqrt{2N^2(\phi_3) - L^2(\phi_1)}, \quad \omega_2 = (\omega_0 - i\gamma_0). \tag{7}$$

Notably, three eigenvalues coalesce when the term under the square root vanishes. The EL3s obtained from Supplementary Equation 6 are plotted in Fig. 2b in the main text. Herein, $\mathcal{D}$ contains the information of both EP2s and EP3s. To further distinguish EP3s from EP2s, we need to find the exclusive condition for EP3s.

The universal form of the characteristic polynomial $p(\omega) = \det(H_{3b} - \omega\boldsymbol{I})$ is

$$p(\omega) = \omega^3 - \mathrm{tr}[H_{3b}]\omega^2 + \frac{(\mathrm{tr}[H_{3b}])^2 - \mathrm{tr}[H_{3b}^2]}{2}\omega - \det\,[H_{3b}], \tag{8}$$

and the corresponding discriminant is

$$\mathcal{D} = -\frac{1}{27}[4\xi^3 + \nu^2], \tag{9}$$

where ξ and ν are both complex-valued constraints when symmetry is absent,

$$\xi_{3b} = \frac{1}{2}(\mathrm{tr}[H_{3b}]^2 - 3\mathrm{tr}[H_{3b}^2]), \tag{10}$$

$$\nu_{3b} = \frac{1}{2}(54\det[H_{3b}] - 5\mathrm{tr}[H_{3b}]^3 + 9\mathrm{tr}[H_{3b}]\mathrm{tr}[H_{3b}^2]). \tag{11}$$

The subscript “3b” denotes the three-band case. When $N = 3$, Supplementary Equation 2 is further simplified as $\mathcal{D} = -\mathcal{R}[p, p^{(1)}]$. The other two resultants are also defined

$$\mathcal{R}[p, p^{(2)}] = \det[\mathrm{Syl}(p, p^{(2)})] = \frac{1}{16}(54\det[H_{3b}] - 5\mathrm{tr}[H_{3b}]^3 + 9\mathrm{tr}[H]\mathrm{tr}[H_{3b}^2]). \tag{12}$$

$$\mathcal{R}[p^{(1)}, p^{(2)}] = \det[\mathrm{Syl}(p^{(1)}, p^{(2)})] = \frac{1}{24}(\mathrm{tr}[H_{3b}]^2 - 3\mathrm{tr}[H_{3b}^2]). \tag{13}$$

We can find

$$\xi_{3b} = \frac{1}{12}\mathcal{R}[p^{(1)}, p^{(2)}], \tag{14}$$

$$\nu_{3b} = \frac{1}{8}\mathcal{R}\left[p, p^{(2)}\right]. \tag{15}$$

Then relation among the three resultants is

$$\mathcal{R}\left[p, p^{(1)}\right] = \frac{1}{27}\left[\frac{1}{432}\mathcal{R}\left[p^{(1)}, p^{(2)}\right]^3 + \frac{1}{64}\mathcal{R}\left[p, p^{(2)}\right]^2\right]. \tag{16}$$

Remarkably, $\mathcal{R}\left[p, p^{(1)}\right]$ vanishes only when $\mathcal{R}\left[p^{(1)}, p^{(2)}\right] = 0$ and $\mathcal{R}\left[p, p^{(2)}\right] = 0$. Since the EP2s are determined by $\mathcal{R}\left[p, p^{(1)}\right] = 0$, either $\mathcal{R}\left[p^{(1)}, p^{(2)}\right] = 0$ or $\mathcal{R}\left[p, p^{(2)}\right] = 0$ fails to detect any EP2s. In a similar fashion, $\mathcal{R}\left[p^{(1)}, p^{(2)}\right]$ and $\mathcal{R}\left[p, p^{(2)}\right]$ can pinpoint EP3s while ignoring EP2s.

Supplementary Note 2 Retrieval of eigenfrequencies

The eigenfrequencies are obtained from the measured response spectra by utilizing the Green's function[1,2], given by

$$\overleftrightarrow{G}(\omega) = \sum_{j=1}^{3} \frac{\left|\psi_j^R\right\rangle\left\langle\psi_j^L\right|}{\omega - \omega_j}, \tag{17}$$

where ω_j denotes the eigenfrequency with j labeling the eigenstates. The normalized biorthogonal right and left eigenvectors are respectively represented by $\left|\psi_j^R\right\rangle$ and $\left\langle\psi_j^L\right|$. Since the Hamiltonian in our work is reciprocal, we have $\left\langle\psi_j^L\right| = \left|\psi_j^R\right\rangle^T$. Besides, the pressure responses measured from three coupled acoustic cavities satisfy

$$P(\omega) = \left\langle m\middle|\overleftrightarrow{G}(\omega)\middle|s\right\rangle, \tag{18}$$

wherein $|s\rangle$ and $|m\rangle$ are 3×1 column vectors, which represent the source and microphone positions, respectively. In our experiment, the source pumps at cavity B, which is represented as $|s\rangle = (0 \quad 1 \quad 0)^T$. The pressure responses are measured at the central position of three cavities, thus $|m\rangle$ are $(1 \quad 0 \quad 0)^T$, $(0 \quad 1 \quad 0)^T$, and $(0 \quad 0 \quad 1)^T$ for cavities A, B, and C, respectively. Then the parameters of the Hamiltonian can be retrieved from the measured pressure response spectrum by applying a genetic algorithm. Some fitting results are selected and plotted in Supplementary Figure 1.

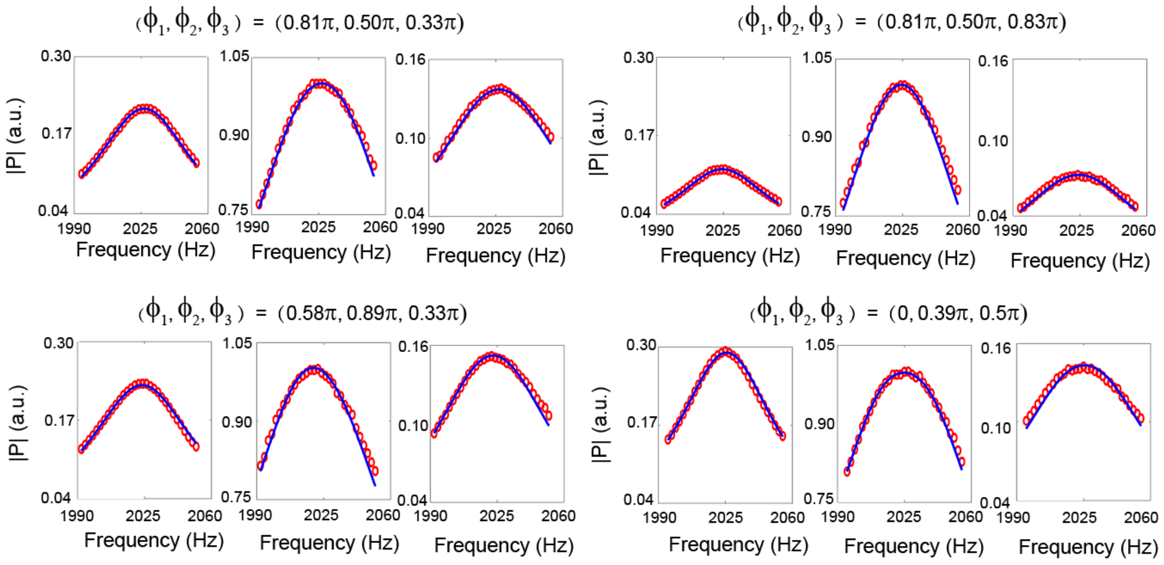

Supplementary Figure 1 | Selected results of the measured and retrieval acoustic pressure response spectra. Red open circles are experimentally attained data. Blue curves are fitted by applying the Green's function method. Good agreement between the two indicates the validity of our fitting approach.

Supplementary Note 3 The multiplicity of EP3s

The resultant field $\Lambda(\vec{\phi}) = \eta + i\zeta$ vanishes at EP3s, which corresponds to the intersections $\eta = 0$ and $\zeta = 0$, as shown in Fig. 4b of the main text. Hence the intersection multiplicity can be treated as the multiplicity of the EP3. For convenience, herein our Hamiltonian H_{3b} is divided by a scaler factor $A = 42.91$, then η and ζ are

$$\eta = -12\begin{bmatrix} 0.81(\delta_M + \cos^2\phi_2)^2 + 6(-1 + 0.62\cos^2\phi_3)^2 \\ -6(0.50\sin^2\phi_1 - 1 + \delta_L)^2 \end{bmatrix}, \quad (19)$$

$$\zeta = -14.4(\delta_M + \cos^2\phi_2)\begin{bmatrix} 18(0.50\sin^2\phi_1 - 1 + \delta_L)^2 + \\ 9(-1 + 0.62\cos^2\phi_3)^2 + 0.81(\delta_M + \cos^2\phi_2)^2 \end{bmatrix}. \quad (20)$$

η and ζ are real when δ_L, $\delta_M \in \mathbb{R}$, which can also be understood from the symmetry perspective. Note that $\zeta = 0$ can be deduced from

$$\delta_M + \cos^2\phi_2 = 0. \quad (21)$$

This implies that when $\delta_M > 0$, no EP3 exists. When $\delta_M < 0$, the EP3s appear around $\phi_2 = \pm\pi/2$. From Supplementary Equation 19, 21, $\eta = 0$ can be derived from

$$[0.50\sin^2\phi_1 - 0.62\cos^2\phi_3 + \delta_L][2 - 0.50\sin^2\phi_1 - 0.62\cos^2\phi_3 - \delta_L] = 0. \quad (22)$$

Concerning our current experimental setup, the location of EL3s is given by

$$0.50\sin^2\phi_1 - 0.62\cos^2\phi_3 = -\delta_L, \quad (23)$$

Then η and ζ vanish when

$$\delta_M + \cos^2\phi_2 = 0, \quad (24)$$

$$0.50\sin^2\phi_1 - 0.62\cos^2\phi_3 + \delta_L = 0. \quad (25)$$

According to Bezout's theorem, the number of intersection points with multiplicity is four in both $\phi_1\phi_2$ and $\phi_2\phi_3$ planes.

Note that when $\delta_M = \delta_L = 0$, we only find a single TP at $\left(0, \frac{\pi}{2}, \frac{\pi}{2}\right)$. We further expand ϕ_1, ϕ_2, ϕ_3 near $\left(0, \frac{\pi}{2}, \frac{\pi}{2}\right)$ as $\sin^2\phi_1 \cong q_1^2, \cos^2\phi_2 \cong q_2^2, \cos^2\phi_3 \cong q_3^2$, then

$$q_2^2 = 0, \quad (26)$$

$$0.50\, q_1^2 - 0.62\, q_3^2 = 0. \quad (27)$$

Likewise, the multiplicity of the TP at $\left(0, \frac{\pi}{2}, \frac{\pi}{2}\right)$ is also four in both q_2q_3 and q_1q_2 planes. When $\delta_M \neq 0$ and $0.62\cos^2\phi_3 - \delta_L \neq 0$, there are four EP3s falling on the $\phi_1\phi_2$ plane or $\phi_2\phi_3$ plane that cuts the EL3. Take the $\phi_1\phi_2$ plane at $\phi_3 = 0.7\pi$ shown in Fig. 5a of the main text for example. To simplify the discussion, we further expand ϕ_1 and ϕ_2 around the EP3 encircled by the purple closed loop, which arises as the intersection *P* of the curves of $\zeta = 0$ and $\eta = 0$. Then the conditions for the vanishing of ζ and η are rewritten as

$$F_\zeta = q_2, \quad F_\eta = q_1. \quad (28)$$

Obviously, when $q_1 = q_2 = 0$, F_ζ and F_η intersect at $P_{\eta,\zeta}$ with multiplicity $u_{P_{\eta,\zeta}}(F_\eta, F_\zeta) = 1$. Notably, EP3 can also be identified as the intersections of either two out of the three resultants (recall that $\eta = \mathcal{R}[p^{(1)}, p^{(2)}]$, $\zeta = \mathcal{R}[p, p^{(2)}]$, and we further denote $\chi \coloneqq \mathcal{R}[p, p^{(1)}]$ for convenience). The conditions given by $\eta = 0$ and $\zeta = 0$ are already discussed as the resultant field $\Lambda = \eta + i\zeta$. But we still have two more possibilities: $\chi = 0$ and $\zeta = 0$, or $\chi = 0$ and $\eta = 0$, as shown in Figure 5b, c of the main text. We investigate the multiplicities of these two intersections, denoted $P_{\chi,\zeta}$ and $P_{\chi,\eta}$. From Supplementary Equation 2, 9, we can deduce $\chi = \frac{1}{27}\left(\frac{1}{432}\eta^3 + \frac{1}{64}\zeta^2\right)$. Near the EP3, the condition of $\chi = 0$ is

$$F_\chi = \frac{1}{27}\left(\frac{1}{432}q_1^3 + \frac{1}{64}q_2^2\right). \quad (29)$$

Remarkably, the tangent of F_χ, denoted as $m(F_\chi)$, is q_2, which is of multiplicity 2. F_ζ also has one tangent of q_2 with the multiplicity being 1, but $F_\eta = q_1$ does not have any linear term in q_2, leading to $m(F_\eta) = 0$. Then we have

$$u_{P_{\chi,\zeta}}(F_\chi, F_\zeta) = m(F_\chi) + m(F_\zeta) = 3, \tag{30a}$$

$$u_{P_{\chi,\eta}}(F_\chi, F_\eta) = m(F_\chi) + m(F_\eta) = 2, \tag{30b}$$

$$u_{P_{\eta,\zeta}}(F_\eta, F_\zeta) = m(F_\eta) + m(F_\zeta) = 1. \tag{30c}$$

Apparently, only $u_{P_{\eta,\zeta}}(F_\eta, F_\zeta)$ exactly characterize the multiplicity of EP3. This is because F_χ is also the condition of EP2 and has singularities at the positions of EP3s. Hence one cannot use F_χ to characterize the multiplicity of the EP3. In other words, χ can be safely used to determine the location of an EP3 but cannot be utilized to characterize its topology because F_χ is a self-intersection curve[3]. To further demonstrate, we herein consider the topological stability of the EP3. According to Fermion doubling theorem[4], when the two constraints determining the EP2 linearly cross locally, the intersection point is stable because small perturbations in the parameters do not destroy it. However, when the two constraints quadratically touch, the intersection point can disappear when we vary the parameters. From the intersection theory perspective, the former has an intersection multiplicity of 1, and the winding number is also 1. In contrast, the latter has an intersection multiplicity of 2, and the winding number is 0. In terms of symmetry-protected EL3s in our system, we define two different resultant fields $\Lambda' = \chi + i\zeta$ and $\Lambda'' = \chi + i\eta$. As shown in Fig 5 in the main text, when encircling the intersection $P_{\eta,\zeta}$ and $P_{\chi,\zeta}$, one can obtain $\mathcal{W}_\Lambda = 1$ (Fig. 5d) and $\mathcal{W}_{\Lambda'} = 1$ (Fig. 5e), respectively, revealing the stabilities of intersection $P_{\eta,\zeta}$ and $P_{\chi,\zeta}$. However, the instability of $P_{\chi,\eta}$ is evident with $\mathcal{W}_{\Lambda''} = 0$ (Fig. 5f), which coincides with their multiplicities. Note that the curve F_χ contains singularity, though the intersection $P_{\chi,\zeta}$ carries $\mathcal{W}_{\Lambda'} = 1$. Thereby, only $\mathcal{W}_\Lambda = 1$ of intersection $P_{\eta,\zeta}$ characterize the topological stability of the EP3 regardless of the interruption of EP2s.

Supplementary Note 4 Homotopic Classifications

The topology of manifolds with EPs and EP structures can be characterized using homotopy groups[5]. To explain briefly, an N-dimensional manifold can be characterized using homotopy groups π_n with $n \leq N$, where n is the dimension of the sphere. Take $n = 2$ and $N = 3$ as the first example, which corresponds to using 2-spheres (denoted S^2), i.e., common spherical surfaces, to characterize 3-dimensional manifolds $M_{3\text{D}}$, e.g., 3D Bloch bands. This is plotted in Supplementary Figure 2a. Suppose the bands form one topological singularity, e.g., an exceptional point (EP), then the 2-sphere can either enclose the singularity or enclose nothing. The 2-sphere enclosing nothing can shrink to a point, whereas the one enclosing the singularity cannot (because the 2-sphere cannot continuously deform across the singularity). This means that there are at least two inequivalent classes of 2-spheres, so the corresponding homotopy group, $\pi_2(M_{3\text{D}})$ contains more than one element, i.e., it is nontrivial.

Supplementary Figure 2b shows another case, which characterizes a 2-dimensional manifold $M_{2\text{D}}$ with two EPs using 1-sphere, i.e., a circle (without the interior). There are multiple choices of inequivalent 1-spheres, and the plotted cases are: enclosing one EP, both EPs, and nothing. These loops are inequivalent for the same reason that they cannot deform into one another. Hence the first homotopy group $\pi_1(M_{2\text{D}})$ is also nontrivial. (Because the circles are allowed to deform as long as they don't cross the EP, one can find loops to wrap around an EP multiple times, and all these loops are inequivalent, which eventually means, for a single EP, π_1 is isomorphic to the integer group $\mathbb{Z}$. But for the discussion here, we need not go into such details.)

Following this methodology, it can be seen that in some cases, e.g., an EP ring in a 3-dimensional manifold, can be characterized using either π_2 or π_1 (Supplementary Figure 2c), an uninterrupted EP line that extends to infinities only accepts π_1 characterization because one can find no 2-sphere to enclose it (Supplementary Figure 2d). And upon examining the situation of our model, it is rather straightforward that one stays in the eigenvalue manifold, one can find no 2-sphere nor 1-sphere to enclose the EL3 without crossing the ES2. Hence the eigenvalue manifold cannot be directly topologically characterized using π_2 or π_1 and it demands us to go to a different manifold (the resultant fields) to identify its topological

properties.

Lastly, we briefly discuss π_0, i.e., the 0-sphere classification. A 0-sphere is a pair of two points. Consider the case in Supplementary Figure 2e, an EP ring in a 2-dimensional manifold. Obviously, one can find three different inequivalent classes of S^0, so π_0 is nontrivial. Specifically, the green and orange pairs can both shrink to one point but cannot deform continuously to one another because such deformation must cross the EP ring. And the blue pair cannot shrink to a single point. However, to the best of our knowledge, π_0 classification is rarely used in the study of eigenvalue manifolds in physics, and we cannot find any interesting topological effect that directly associates with π_0 invariants.

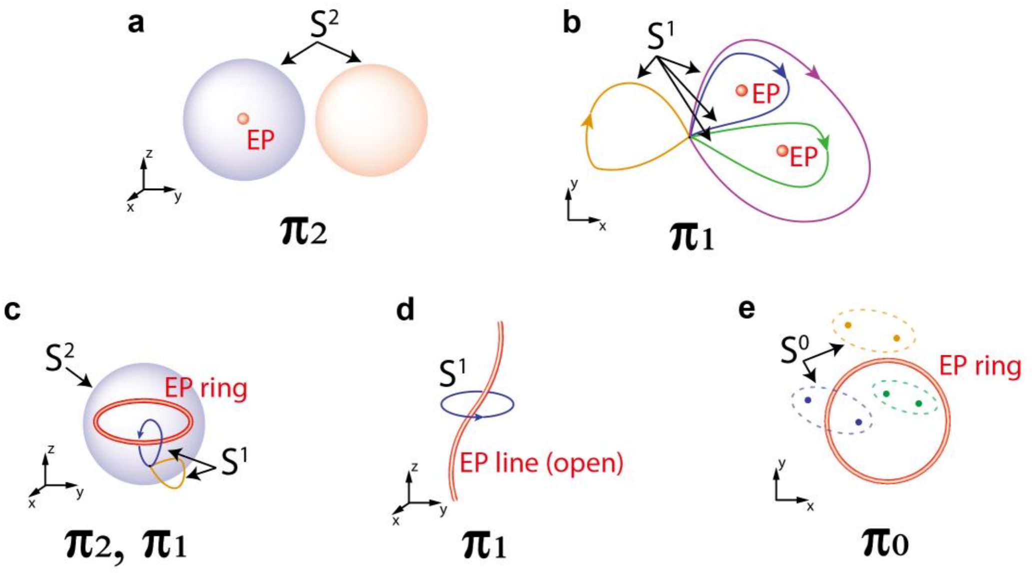

Supplementary Figure 2 | Homotopic classification of manifolds with EPs and EP structures. a, A 3-dimensional manifold $M_{3\mathrm{D}}$ with an EP characterized by π_2. **b,** A 2-dimensional manifold $M_{2\mathrm{D}}$ containing two EPs characterized by π_1. **c,** A 3-dimensional manifold sustaining an EP ring is characterizable by both π_1 and π_2. **d,** An uninterrupted EP line in a 3-dimensional manifold is characterizable only by π_1. **e,** π_0 classification of an EP ring in a 2-dimensional manifold.

Supplementary Note 5 EL2 without PT-symmetry

In our system, the existence of PT-symmetry reduces the codimension from $2(n-1)$ to $n-1$, making the EPn locally stable in the $(n-1)$-dimensional space. When two types of symmetry-preserving perturbations (δ_L and δ_M) are introduced, the PT-symmetry of the Hamiltonian remains, and thus the EL3 is locally stable and protected by the PT-symmetry,

as shown in Fig. 4 of the main text. In contrast, when any symmetry-breaking perturbation is added, e.g., $L(\phi_1) = -60.68(0.50\sin^2\phi_1 - 1 + \delta_L')$ with $\delta_L' = 0.1i$, the EL3 disappears since PT-symmetry is broken. Then there only exists the EL2 in the 3D parameter space, as shown in Supplementary Figure 3.

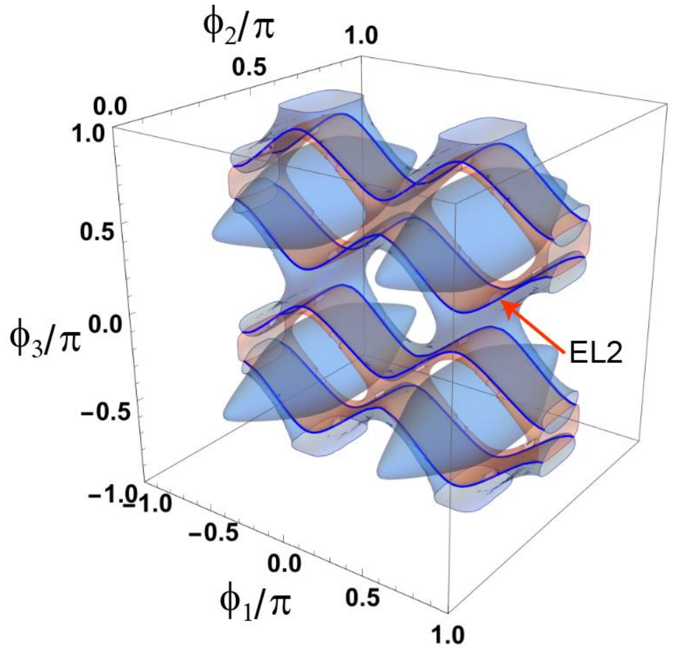

Supplementary Figure 3 | EL2 in the 3D parameter space when PT-symmetry is broken. The blue (orange) surfaces correspond to $\mathrm{Re}(\mathcal{D}) = 0$ [$\mathrm{Im}(\mathcal{D}) = 0$]. The EL2 (blue solid lines) occur as the intersections of the blue and red surfaces.

Supplementary References

1. Ding, K., Ma, G., Xiao, M., Zhang, Z. Q. & Chan, C. T. Emergence, Coalescence, and Topological Properties of Multiple Exceptional Points and Their Experimental Realization. *Physical Review X* **6**, 021007 (2016).
2. Tang, W. *et al.* Exceptional nexus with a hybrid topological invariant. *Science* **370**, 1077–1080 (2020).
3. Delplace, P., Yoshida, T. & Hatsugai, Y. Symmetry-Protected Multifold Exceptional Points and Their Topological Characterization. *Phys. Rev. Lett.* **127**, 186602 (2021).
4. Yang, Z., Schnyder, A. P., Hu, J. & Chiu, C.-K. Fermion Doubling Theorems in Two-Dimensional Non-

Hermitian Systems for Fermi Points and Exceptional Points. *Phys. Rev. Lett.* **126**, 086401 (2021).

5. Spanier, E. H. *Algebraic Topology*. (Springer New York, 1981). doi:10.1007/978-1-4684-9322-1.
